# Supplementary material for: Hyperglycemia and bladder cancer prognosis in a Finnish population-based cohort
Source: PLoS One. 2025 Dec 12;20(12):e0338215. doi: 10.1371/journal.pone.0338215 (PMC12700417; doi:10.1371/journal.pone.0338215)
Supplement: Attachment — Submitted filename: Review Comments.pdf [file pone.0338215.s002.pdf]

The study includes a large cohort of 14,638 BCa patients diagnosed in Finland between 1995 and 2012. Utilizing data from three national databases, the study evaluates the impact of diabetes, measured through blood glucose levels and HbA1c, on BCa-specific and overall mortality using multivariable Cox regression models. The findings indicate that diabetic blood glucose levels are associated with worse BCa-specific and overall survival, with the increased risk being slightly mitigated among those using antidiabetic medication. The study highlights the need for further research to understand the mechanisms behind these associations and the potential implications for clinical management of BCa patients with diabetes.

Here are my questions based on the article:

1. How did the researchers ensure the completeness and accuracy of the data obtained from the three national databases, and what measures were taken to address any potential data gaps or inconsistencies?
2. What criteria were used to classify participants' glycemic status, and how did the study account for variations in blood glucose levels and HbA1c measurements over time?
3. How did the use of antidiabetic medication influence the survival outcomes of BCa patients, and what potential mechanisms might explain the observed differences in mortality risk between medication users and non-users?
4. What confounding factors were considered in the multivariable Cox regression models, and how did the study address potential confounders such as smoking and comorbidities?
5. How did the associations between diabetic glycemic status and BCa-specific mortality differ by gender and cancer stage, and what implications do these findings have for personalized treatment strategies in BCa patients with diabetes?

Here are the grammatical and phrasing errors found in the provided document along with corrections:

1. Location: Abstract (Lines 21-22)
   - Original: "Diabetes might increase a risk of bladder cancer (BCa) incidence and bladder cancer specific death."
   - Correction: "Diabetes might increase the risk of bladder cancer (BCa) incidence and bladder cancer-specific death."

2. Location: Abstract (Lines 27-28)
   - Original: "Three databases were used to obtain information on BCa, diabetes and comorbidities."
   - Correction: "Three databases were used to obtain information on BCa, diabetes, and comorbidities."

3. Location: Abstract (Lines 30-31)
   - Original: "During median follow-up of 4.3 years after BCa diagnosis 3,582 (24.5 %) participants died of BCa."
   - Correction: "During a median follow-up of 4.3 years after BCa diagnosis, 3,582 (24.5%) participants died of BCa."

4. Location: Abstract (Line 33)
   - Original: "The risk increase was slightly lower among hyperglycemic DM medication users (HR 1.43 (0,68–3.02) than non-users HR 1.80 (1.13–2.85)."
   - Correction: "The risk increase was slightly lower among hyperglycemic DM medication users (HR

1.43 (0.68–3.02)) than non-users (HR 1.80 (1.13–2.85))."

5. Location: Abstract (Line 37)
   - Original: "This was observed in both genders."
   - Correction: "This was observed in both men and women."

6. Location: Introduction (Line 43)
   - Original: "Bladder cancer (BCa) is the ninth most common cancer worldwide [1]. Well-known risk factors are smoking, male gender, living in developed countries and chemical exposure [1,2]."
   - Correction: "Bladder cancer (BCa) is the ninth most common cancer worldwide [1]. Well-known risk factors include smoking, being male, living in developed countries, and chemical exposure [1,2]."

7. Location: Introduction (Line 50)
   - Original: "These presumably support growth of cancer cells by offering energy and growth factors for rapid cell growth."
   - Correction: "These presumably support the growth of cancer cells by providing energy and growth factors for rapid cell growth."

8. Location: Results (Line 156)
   - Original: "No clear differences in Charlson co-morbidity score or prevalence of COPD were observed by medication use or blood glucose level."
   - Correction: "No clear differences in Charlson comorbidity score or prevalence of COPD were observed by medication use or blood glucose level."

9. Location: Results (Line 160)
   - Original: "DM medication users had similar overall mortality than non-users, while hyperglycemic participants had higher overall mortality than normoglycemic participants."
   - Correction: "DM medication users had similar overall mortality to non-users, while hyperglycemic participants had higher overall mortality than normoglycemic participants."

10. Location: Results (Line 174)
   - Original: "The risk increase was slightly lower among hyperglycemic DM medication users HR 1.43 (0,68–3.02) than non-users HR 1.80 (1.13–2.85) but remained elevated compared to normoglycemia in both groups."
   - Correction: "The risk increase was slightly lower among hyperglycemic DM medication users (HR 1.43 (0.68–3.02)) than non-users (HR 1.80 (1.13–2.85)) but remained elevated compared to normoglycemia in both groups."

11. Location: Discussion (Line 219)
   - Original: "Expectedly, also overall mortality was higher in diabetics compared to non-diabetics."
   - Correction: "As expected, overall mortality was also higher in diabetics compared to non-diabetics."

12. Location: Discussion (Line 240)
   - Original: "Therefore, they might be treated in less aggressive manner."

- Correction: "Therefore, they might be treated in a less aggressive manner."

13. Location: Discussion (Line 258)
  - Original: "For our knowledge only one study has previously reported increased risk of BCa-specific death among women with diabetes."
  - Correction: "To our knowledge, only one study has previously reported an increased risk of BCa-specific death among women with diabetes."

14. Location: Discussion (Line 265)
  - Original: "To decrease this limitation, we adjusted the analysis for records of COPD and smoking from the HILMO database, but the information especially on smoking was severely limited."
  - Correction: "To mitigate this limitation, we adjusted the analysis for records of COPD and smoking from the HILMO database, but the information on smoking was particularly limited."

15. Location: Conclusions (Line 281)
  - Original: "whereas antidiabetic medication use may mitigate the risk increase to small degree."
  - Correction: "whereas antidiabetic medication use may mitigate the risk increase to a small degree."
