# Supplementary material for: Hyperglycemia and bladder cancer prognosis in a Finnish population-based cohort
Source: PLoS One. 2025 Dec 12;20(12):e0338215. doi: 10.1371/journal.pone.0338215 (PMC12700417; doi:10.1371/journal.pone.0338215)
Supplement: Attachment — Submitted filename: Response to Reviewers.docx [file pone.0338215.s001.docx]

Dear Editor,

Thank you for the opportunity to revise our manuscript according to Your comments.

Answers to editor´s and reviewer´s comments are attached below.

Changes also are visible in “track changes” version of manuscript.

Journal Requirements:

When submitting your revision, we need you to address these additional requirements.

1. Please ensure that your manuscript meets PLOS ONE's style requirements, including those for file naming. The PLOS ONE style templates can be found at 
[https://journals.plos.org/plosone/s/file?id=wjVg/PLOSOne_formatting_sample_main_body.pdf](https://journals.plos.org/plosone/s/file?id=wjVg/PLOSOne_formatting_sample_main_body.pdf) and 
[https://journals.plos.org/plosone/s/file?id=ba62/PLOSOne_formatting_sample_title_authors_affiliations.pdf](https://journals.plos.org/plosone/s/file?id=ba62/PLOSOne_formatting_sample_title_authors_affiliations.pdf).

Answer: Manuscript is now edited to meet PLOS ONE´s style requirements.

2. You indicated that ethical approval was not necessary for your study. We understand that the framework for ethical oversight requirements for studies of this type may differ depending on the setting and we would appreciate some further clarification regarding your research. Could you please provide further details on why your study is exempt from the need for approval and confirmation from your institutional review board or research ethics committee (e.g., in the form of a letter or email correspondence) that ethics review was not necessary for this study? Please include a copy of the correspondence as an ""Other"" file.

Answer: In compliance with Finnish law on medical research (9.4.1999/488) Institutional review board approval can be waived for studies based entirely on routinely collected registry data. This text is added to end of the Material and methods section.

3. Thank you for stating the following financial disclosure: 
"The authors received no specific funding for this work.
The funders had no role in study design, data collection and analysis, decision to publish, or preparation of the manuscript."

At this time, please address the following queries:

a) Please clarify the sources of funding (financial or material support) for your study. List the grants or organizations that supported your study, including funding received from your institution. 
b) State what role the funders took in the study. If the funders had no role in your study, please state: “The funders had no role in study design, data collection and analysis, decision to publish, or preparation of the manuscript.”
c) If any authors received a salary from any of your funders, please state which authors and which funders.
d) If you did not receive any funding for this study, please state: “The authors received no specific funding for this work.”

Please include your amended statements within your cover letter; we will change the online submission form on your behalf.

Answer: The statement “The authors received no specific funding for this work.” is added to the cover letter.

4. In your Data Availability statement, you have not specified where the minimal data set underlying the results described in your manuscript can be found. PLOS defines a study's minimal data set as the underlying data used to reach the conclusions drawn in the manuscript and any additional data required to replicate the reported study findings in their entirety. All PLOS journals require that the minimal data set be made fully available. For more information about our data policy, please see [http://journals.plos.org/plosone/s/data-availability](http://journals.plos.org/plosone/s/data-availability).

Upon re-submitting your revised manuscript, please upload your study’s minimal underlying data set as either Supporting Information files or to a stable, public repository and include the relevant URLs, DOIs, or accession numbers within your revised cover letter. For a list of acceptable repositories, please see [http://journals.plos.org/plosone/s/data-availability#loc-recommended-repositories](http://journals.plos.org/plosone/s/data-availability#loc-recommended-repositories). Any potentially identifying patient information must be fully anonymized.

Important: If there are ethical or legal restrictions to sharing your data publicly, please explain these restrictions in detail. Please see our guidelines for more information on what we consider unacceptable restrictions to publicly sharing data: [http://journals.plos.org/plosone/s/data-availability#loc-unacceptable-data-access-restrictions](http://journals.plos.org/plosone/s/data-availability#loc-unacceptable-data-access-restrictions). Note that it is not acceptable for the authors to be the sole named individuals responsible for ensuring data access.

We will update your Data Availability statement to reflect the information you provide in your cover letter.

Answer: Following text is added to cover letter: Data availability statement: The datasets generated and analyzed during the current study are not publicly available as it includes personal level data. Data is gathered from several governmental registries and specific permission from national authority FINDATA is needed to access the data.

5. Please amend either the abstract on the online submission form (via Edit Submission) or the abstract in the manuscript so that they are identical.

Answer: Abstract is copied from manuscript to the online submission form so they are identical.

6. We note you have included a table to which you do not refer in the text of your manuscript. Please ensure that you refer to Table 5 in your text; if accepted, production will need this reference to link the reader to the Table.

Answer: The table is now in right position and referred in the text.

7. Please include captions for your Supporting Information files at the end of your manuscript, and update any in-text citations to match accordingly. Please see our Supporting Information guidelines for more information: [http://journals.plos.org/plosone/s/supporting-information](http://journals.plos.org/plosone/s/supporting-information).

Answer: The Supplementary Table S1 was removed from the manuscript. The table is however referred to in the text, and is found in the reference list (14. The Anatomical Therapeutic Chemical (ATC) classification system. https://www.whocc.no/atc_ddd_index/).

Additional Editor Comments:

I revised the paper entitled "Diabetes and bladder cancer prognosis in a Finnish population-based cohort". It was a cohort study based on Finnish diseases registry aimed to evaluate the association between diabetes and the incidence on bladder cancer.
The topic is interesting and data were robust. Statistical analysis is strong and appropriate.
observations:
- Describe better the HILMO;

Answer: HILMO is described in Material and methods section page 4, line 80. Text was added to line 80: “at every medical treatment facility” for clarification.

- Describe how missing data were handled;

Answer: Only those participants with sufficient information were included in the analysis. Total 14,638 participants were included. Fasting glucose levels were available for a subgroup of 624 participants.

We have added this clarification to Materials and Methods, Statistical analysis section.

“In Cox regression analyses, ~~diabetes~~ glycemic status before BCa diagnosis was analyzed as a time-fixed variable taking into account all glucose and HbA1C measurements, diagnoses and drug purchases occurring between 1995 and the year of BCa diagnosis. Glycemic status after BCa diagnosis was analyzed as time-dependent variable, where the status was updated separately for each follow-up year. As we used time-varying covariates for the analysis, checking for validity of the proportional hazards assumption over time was not necessary. ~~Participants without any measurement of fasting glucose or HbA1c levels were not included in the analysis.~~

~~Participants remained as unexposed until the first year of either recorded DM diagnosis or antidiabetic drug purchase.~~ Same model adjustments were used as for analysis of pre-diagnosis DM.

The time-dependent analysis was separately made for fasting glucose and HbA1c levels. For years with no blood glucose measurements available, we used the latest available blood glucose level. ~~Participants without any measurement of fasting glucose or HbA1c levels in given year during the follow-up were included in the analysis as a separate group.~~ Participants without any measurement of fasting glucose or HbA1c levels were not included in the analysis.”

- Why the last followup was set at 2014?

Answer: The information on deaths was available up until March 2014.

- Smoke is one of most important risk factor for bladder cancer, so Authors should correct also the analysis with this variable

Answer: The information on tobacco smoking was not available from the registries and could not be used as a variable in the analysis.

- Among limitations, Authors should consider the occupational exposure and the environmental exposure as risk factors that cannot be considered in this study.

Answer: Text was added to page 13 line 251: (chemical exposure, mostly in occupational exposure is known risk factor for BCa)

Reviewers' comments:

Reviewer's Responses to Questions

**Comments to the Author**

1. Is the manuscript technically sound, and do the data support the conclusions?

The manuscript must describe a technically sound piece of scientific research with data that supports the conclusions. Experiments must have been conducted rigorously, with appropriate controls, replication, and sample sizes. The conclusions must be drawn appropriately based on the data presented.

Reviewer #1: Yes

Reviewer #2: Yes

2. Has the statistical analysis been performed appropriately and rigorously?

Reviewer #1: No

Reviewer #2: Yes

3. Have the authors made all data underlying the findings in their manuscript fully available?

The [PLOS Data policy](http://www.plosone.org/static/policies.action#sharing) requires authors to make all data underlying the findings described in their manuscript fully available without restriction, with rare exception (please refer to the Data Availability Statement in the manuscript PDF file). The data should be provided as part of the manuscript or its supporting information, or deposited to a public repository. For example, in addition to summary statistics, the data points behind means, medians and variance measures should be available. If there are restrictions on publicly sharing data—e.g. participant privacy or use of data from a third party—those must be specified.

Reviewer #1: No

Reviewer #2: Yes

4. Is the manuscript presented in an intelligible fashion and written in standard English?

PLOS ONE does not copyedit accepted manuscripts, so the language in submitted articles must be clear, correct, and unambiguous. Any typographical or grammatical errors should be corrected at revision, so please note any specific errors here.

Reviewer #1: Yes

Reviewer #2: Yes

5. Review Comments to the Author

Please use the space provided to explain your answers to the questions above. You may also include additional comments for the author, including concerns about dual publication, research ethics, or publication ethics. (Please upload your review as an attachment if it exceeds 20,000 characters)

Reviewer #1:

This manuscript addresses the association of the duration and timing of diabetes with bladder cancer-specific and overall mortality in a large register-based cohort study conducted in Finland, including the role of diabetic glycemic status and use of antidiabetic medication. This study is of interest but there are several points that need further clarification or need to be adjusted. English language is not always correct and should be checked.

Answer: following changes are made after the language check:

Introduction:

According to a meta-analysis of 36 studies, ~~showed that~~ diabetes was associated with an increased risk of BCa in men [6].

~~“We performed a population-based cohort study to explore risk associations between diabetes and BCa-specific mortality. We hypothesized that diabetics would have a worse disease-specific survival.”~~ was replaced with “We conducted a population-based cohort study to investigate the associations between diabetes and BCa-specific mortality, hypothesizing that individuals with diabetes would experience worse disease-specific survival.”

Material and methods:

~~“Ten cases who were under 20 years of age at diagnosis were excluded, additionally 182 cases were excluded in whom BCa diagnosis was made at the time of death or after it, and who did not die of BCa.”~~ was replaced with “Ten cases diagnosed under the age of 20 were excluded. Additionally, 182 cases were excluded in which the BCa diagnosis was made at the time of death or thereafter, and these individuals did not die of BCa.”

Results:

The proportion of men was higher in the diabetic group ~~than in~~ compared to non-diabetics

Higher post-diagnostic HbA1c levels ~~had~~ showed a similar association with increased risk of BCa death HR 1.50 (0.88–2.56) and overall survival HR 1.43 (1.06–1.93), with the ~~but~~ result being ~~was~~ statistically significant only for overall survival (Table ~~4~~3).

~~Also,~~ Additionally, the risk increase for bladder cancer death was slightly higher than in main analysis, HR 1.96 (0.86–4.47).

Major comments
- Abstract, Results and Discussion: In the Discussion it is reported that "A modest increasing risk trend for BCa death was observed by duration of antidiabetic medication use before bladder cancer diagnosis; the association was strongest in those
who had anti-DM medication use for longer than 7 years before BCa diagnosis. No clear risk trends were observed by duration of medication use after BCa diagnosis." However, these results for duration and timing of diabetes are not described in the Abstract, Results and Tables and should be added. Results for timing are also not addressed in the Abstract.

Answer: We chose to concentrate on prognostic role of blood glucose level in this manuscript, therefore we have removed the parts o text referring to antidiabetic medication, apart from medication use being a stratification variable.

- Methods, p4: Data were obtained for cases diagnosed from 1995-2012. Endpoint information was only received up until March 2014. Why did you choose to collect this until 2014 and why did you not include more recent endpoint information? This should be motivated in the manuscript. Would it also be possible to obtain endpoint information up until e.g. 2021 and include this in the manuscript?

Answer: The information was available from Statistics Finland only up until March 2014.

- Methods, p5: It is stated that participants with either recorded diagnosis of diabetes or any antidiabetic drug purchases were categorized as diabetic. However, in the tables participants are only classified based on antidiabetic medicine use and post-diagnosis blood glucose level. Did you also analyse the participants with information on antidiabetic medicine use and/or a recorded diagnosis of diabetes?

Answer: For the sake of clarity, we chose to concentrate on prognostic role of blood glucose level in this manuscript, therefore we have removed the parts of text referring to antidiabetic medication, apart from medication use being a stratification variable. Results on antidiabetic medication will be reported in a separate manuscript.

- Methods, p6: Since data on primary treatment is missing for 20% of the patients and data on tumor extent for 26% of the patients, how did you deal with this in your statistical analysis? These factors are used as adjustment variables. Did you conduct a complete case analysis (not preferred) or did you use multiple imputation (preferred)? This should be described and multiple imputation should be considered when these data are missing at random.

Answer: Thank you for raising this point. We performed a sensitivity analysis where cases with missing information on primary treatment or tumor stage were excluded. This left us with a cohort of 10,854 patients. In this analysis post-diagnostic diabetic blood glucose was no longer associated with cancer-specific survival: HR 0.97 95% CI 0.67-1.41, nor overall survival HR 1.06, 95% CI 0.82-1.37.

This suggests that cases with missing information on stage may indeed affect the outcome. We have added this sensitivity analysis and findings to

Materials & Methods:

“We performed a sensitivity analyses limited to 10,854 cases with available information on tumor extent at diagnosis. Cancer-specific survival and overall survival were estimated by post-diagnostic glucose level.”

Results (Sensitivity analysis):

“The association between post-diagnostic glucose level and cancer-specific or overall mortality was no longer significant in sensitivity analysis limited to cases with available information on tumor extent at diagnosis, HR 0.97 (95% CI 0.67-1.41) and HR 1.06, (95% CI 0.82-1.37), respectively.”

and Discussion

“Further, information on tumor extent on diagnosis was available only for 74% of the cohort. Limiting the analysis to cases with this information available abolished the risk association between post-diagnostic glucose level and mortality. This suggests that cases with missing information may cause bias in our results.”

- Results, p8 / Tables: Number of deaths are expressed per 1,000 participants in the Results and the Tables but this does not take into account potential differences in follow-up time. This should be replaced by number of deaths/1,000 person-years (or any other number of person-years).

Answer: We have replaced the numbers to be /1,000 person years as requested

- Discussion: Studies cites in this meta-analysis ([https://downloads.hindawi.com/journals/jo/2022/1997507.pdf](https://downloads.hindawi.com/journals/jo/2022/1997507.pdf)) should be referenced and results should be compared an differences between studies should be discussed.

Answer: The meta-analysis is now referenced in the manuscript (17. Dong Lv, Ying Xiang, Tao Song, et al. Diabetes Is a Risk Factor for the Prognosis of Patients with Bladder Cancer: A Meta-Analysis. J Oncol. 2022 Sep 26;2022:1997507. doi: 10.1155/2022/1997507.)

Minor comments
- Abstract: In the objective, please replace 'whether they correlate with' with 'whether they are associated with'.

Answer: “correlate” verb is replaced with “are associated”.

- Methods:
- In the statistical analysis, please add whether you checked the proportional hazards assumption and how this was done.

Answer: We used time-varying covariates for the analysis; therefore checking for validity of proportional hazards assumption over time was not necessary. This has been added to the text to Material and Methods (statistical analysis) section.

- Why was the time metric in years and months and not in days?

Answer: Median survival for even invasive bladder cancer is measured in years, therefore having years and months as the time metric was the suitable choice.

- Results: Table 2 is about prediagnostic, not about postdiagnostic glucose levels. Table 3 is about postdiagnostics, not prediagnostic glucose levels. Table 4 is described before Table 3. Table 5 is not referenced in the Results. Please adjust. In Table 5, which subgroup results are in the first rows (lower total numbers than for e.g. men and women)? Results for duration should be added.
- Tables 2-5: Adjustment factors should be reported in the footnote of the tables.

Answer: The tables are now adjusted and referenced in the text. Duration was not included in the analysis. Adjustment factors are now reported in the footnote of the tables.

- Discussion: It is stated that "Information on tobacco smoking was insufficient. To decrease this limitation, we adjusted the analysis for records of COPD and smoking from the HILMO database, but the information especially on smoking was severely limited." Results of this analysis are not reported and should be added.

Answer: The information on tobacco smoking was based on registere diagnosis, thu severely limited and underreported. TARKISTETAAN OLIKO COPD VAKIOINNEISSA MUKANA

Reviewer #2:

Some questions:

- diabetes with ICD-10 codes E10 and E11 were included. Why other specific types like single gene (E13) and unspecified (E14) were excluded? Miscoding is common.

Answer: That is true. But in our opinion, these two chosen codes are used in Finland, and these other specific types are rare and could cause bias as etiology of these specific types are different from “normal” diabetes.

- diabetes duration. Calculations is done “diabetes before” and “after BCa”. Was the overall duration of diabetes available?

Answer: No. Overall duration of diabetes was unfortunately not available from the registers.

- cut-off points for glucose (is it blood or plasma?) is not given.

Answer: In page 6, line 112 cut-off points are given in following text: “Men were defined as normoglycaemic, or diabetic separately for each follow-up years (<53 to ≥53 mmol/mol, and ≤7 to >7 mmol/l) based on yearly mean HbA1c or median fasting glucose.”

- HbA1c cut-off point 53 mmol/mol is quite high. Although it is the recommended treatment level on current guidelines for sufficient long term glucose targets, number of type 2 patients achieves subnormal HbA1c levels for years. And even at least 10-15% of type 1 patients. Were calculations done using other cut-off like HbA1c 42 mmol/l (diabetes diagnosis level) or 48 mmol/mol. Did it change findings?

Answer: Initially we made calculations and comparisons with three HbA1c groups: < 42 mmol/l, 42 to 53 mmol/l and > 53 mmol/l. The group of 42 to 53 mmol/l was too small to be included in the analysis. 53 mmol/mol is the recommended treatment level and was chosen as the cut-off point.

- mean yearly glucose and HbA1c was used for statistics. How many yearly points were available? It may be possible to calculate “yearly glucose load” either using glucose (time duration x glucose) or HbA1c (time x HbA1c). This gives estimate of long-term glucose load, especially reflecting urine glucose and possible local effects.

Answer: Some participants had up to 17 glucose points in some years and others had zero. It is not possible to calculate yearly glucose load as time duration was not available.

Study hypothesis is relevant
No concerns on statistics
Tables ok. Shows clearly that some groups are quite small
Discussion ok. Possibly include some explanation for previous questions/suggestions.
