# Supplementary material for: Hyperglycemia and bladder cancer prognosis in a Finnish population-based cohort
Source: PLoS One. 2025 Dec 12;20(12):e0338215. doi: 10.1371/journal.pone.0338215 (PMC12700417; doi:10.1371/journal.pone.0338215)
Supplement: Attachment — Submitted filename: Response_to_Reviewers_auresp_1.docx [file pone.0338215.s003.docx]

PONE-D-24-16036

Diabetes and bladder cancer prognosis in a Finnish population-based cohort

PLOS ONE


Dear editor,
Thank You for your interest in our research. We have thoroughly considered the reviewer´s remarks and provide below detailed responses to each comment, along with the revised version of our manuscript. We hope that the revised version of our manuscript will be suitable for publication in PLOS ONE.

Journal Requirements:

When submitting your revision, we need you to address these additional requirements.

1. Please ensure that your manuscript meets PLOS ONE's style requirements, including those for file naming. The PLOS ONE style templates can be found at 
<https://journals.plos.org/plosone/s/file?id=wjVg/PLOSOne_formatting_sample_main_body.pdf> and 
<https://journals.plos.org/plosone/s/file?id=ba62/PLOSOne_formatting_sample_title_authors_affiliations.pdf>

A: Style requirements were checked and references were edited accordingly. File names have been renamed according to PLOS ONE requirements.

2. Thank you for stating the following in the Competing Interests section: 
"Teuvo LJ Tammela: consultant fees from Astellas, Bayer and Roche.

Teemu J Murtola: consultant fees from Novartis, Astellas, Janssen Cilag, Amgen and Recordati. Lecture fees from Ferring, Novartis, Sanofi, Bayer, Roche, Pfizer, Ipsen, Astellas, Amgen and Janssen Cilag. Research funding from Bayer.
Other authors declare no conflicts of interest."

Please confirm that this does not alter your adherence to all PLOS ONE policies on sharing data and materials, by including the following statement: "This does not alter our adherence to PLOS ONE policies on sharing data and materials.” (as detailed online in our guide for authors <http://journals.plos.org/plosone/s/competing-interests>).  If there are restrictions on sharing of data and/or materials, please state these. Please note that we cannot proceed with consideration of your article until this information has been declared.

Please include your updated Competing Interests statement in your cover letter; we will change the online submission form on your behalf.

A: We have added the required declaration both to the manuscript (competing interests section) and the cover letter.

3. We note that you have indicated that there are restrictions to data sharing for this study. PLOS only allows data to be available upon request if there are legal or ethical restrictions on sharing data publicly. For more information on unacceptable data access restrictions, please see <http://journals.plos.org/plosone/s/data-availability#loc-unacceptable-data-access-restrictions>.

Before we proceed with your manuscript, please address the following prompts:

a) If there are ethical or legal restrictions on sharing a de-identified data set, please explain them in detail (e.g., data contain potentially identifying or sensitive patient information, data are owned by a third-party organization, etc.) and who has imposed them (e.g., a Research Ethics Committee or Institutional Review Board, etc.). Please also provide contact information for a data access committee, ethics committee, or other institutional body to which data requests may be sent.

b) If there are no restrictions, please upload the minimal anonymized data set necessary to replicate your study findings to a stable, public repository and provide us with the relevant URLs, DOIs, or accession numbers. For a list of recommended repositories, please see
<https://journals.plos.org/plosone/s/recommended-repositories>. You also have the option of uploading the data as Supporting Information files, but we would recommend depositing data directly to a data repository if possible.

We will update your Data Availability statement on your behalf to reflect the information you provide.

A: Data availability statement has been edited and revised according to the option A. Changes are made to the cover letter, with the following text:

The study protocol was reviewed and approved by the ethics committee of the Pirkanmaa hospital district, Tampere, Finland, as well as by the administrators of each health care registry used in this study. The study utilized solely routinely collected data from national health-care registries, approvals and consents were obtained from registry keepers, Finnish Institute for Health and Welfare (THL) and Social Insurance Institution of Finland (SII).

In compliance with Finnish law on medical research (9.4.1999/488) IRB approval can be waived for studies based entirely on routinely collected registry data and no approval or waiver of ethics approval are needed, nor granted.

The datasets used in the current study are not publicly available as they contain potentially identifiable personal-level data and are owned by registry keepers of each national registry used in our study. Data can be accessed only with permission from the Finnish national authority FINDATA; https://findata.fi/en/.

Reviewers' comments:

Reviewer's Responses to Questions

**Comments to the Author**

Reviewer #1:

The authors investigate the potential association between diabetes and BCa prognosis, but there are several questions that need to be answered before considering the publication.
1) The use of nationwide registries is commendable, but the specific criteria for inclusion and exclusion of data need to be detailed.

A: We included all recorded, newly diagnosed bladder cancer cases in Finland during the study period. Only exclusions were cases diagnosed before the age of 20 years, and cases diagnosed after the date of death, i.e. autopsy-diagnosed cases. This has been clarified in Materials and Methods, study cohort, 1^st^ paragraph:

“Data was obtained from the Finnish Cancer Registry (FCR) for all newly diagnosed BCa cases in Finland 1995-2012.”

“Ten cases diagnosed under the age of 20 were excluded. Additionally, 182 cases were excluded in which the BCa diagnosis was made at the time of death or thereafter. “

2) The limitations section is brief; a more thorough discussion of potential biases and their impact on the results is needed.

A: We have expanded our Discussion on study limitations:

“There were also limitations to this study. Blood glucose measurements were available only for a subgroup. Information on tobacco smoking is not routinely recorded by national registries, thus the information was insufficient. Smoking may have confounded the results, likely raising the observed BCa mortality among anti-DM medication users, if smoking was more common among them.”

“Furthermore, we did not have information on intravesical treatments, like BCG instillations. However, these are unlikely to differ by anti-DM medication use. We neither had information on diet, physical activity, occupation (chemical exposure, mostly in occupational exposure is known risk factor for BCa) or socioeconomic status that may have served as confounding factors, exaggerating the observed risk differences.”

“Even though using of national registries allowed forming a population-based cohort of BCa cases covering the entire of Finland, it also meant heterogenous data with incomplete information on key variables like smoking or tumor extent. Therefore, further studies are needed with more detailed information to validate our findings.”

3) The study mentions the lack of smoking data but does not discuss how this might affect the results.

A: We now clarify that in the Discussion:

“Information on tobacco smoking is not routinely recorded by national registries, thus the information was insufficient. Smoking may have confounded the results, likely raising the observed BCa mortality among anti-DM medication users, if smoking was more common among them.”

4) The study should discuss whether there are significant differences in the results between men and women.

A: We ran subgroup analyses to test for this, but no significant differences in the results between men and women were observed. We report this in the Results, subgroup analyses-section: “Age and gender did not modify the risk association between diabetic blood glucose level and bladder cancer specific and overall survival (p for interaction > 0.05).”

We also highlight this in the Discussion:

“Based on subgroup analysis, hyperglycemia was a similar risk factor of BCa death both among men and women, suggesting that BCa patients may benefit from hyperglycemia management regardless of their gender. Concordantly, two cohort studies of 2,110,074 participants observed similar difference for BCa mortality by diabetes both among men and women. These studies had larger numbers of women compared to our study population. However, proportion of women with fatal BCa was higher in our study compared to the previous studies: proportion of BCa deaths among women was 28.9% in our study cohort vs. 5.2% and 7.3% in the previous studies [7,18]. This could suggest that our study cohort has more high-risk cases, allowing more statistical power to evaluate BCa-specific survival than previous studies.”

5) More detailed information on drug resistance, including specific mechanisms, should be provided.

A: This is a very broad topic, as multiple mechanisms of resistance have been reported to each antidiabetic drug group. It is also mainly beyond the scope of this article, as we are not able to evaluate such mechanisms in our register-based data.

Nevertheless, this is likely one of the reasons why diabetic glucose levels were observed also among antidiabetic medication users. Therefore, we have added the following paragraph to Discussion, 3^rd^ paragraph:

“Hyperglyemia was observed even among DM medication users, which highlights the challenges of diabetes treatment. Resistance to anti-DM drugs may arise via multiple mechanisms, with impaired insulin sensitivity of peripheral tissues and compromised insulin production in pancreatic beta-cells being the most important. Insensitivity to metformin and targeted drugs like GLP-1 receptor agonists and DPP-4 inhibitors may arise via non-canonical activation of their targets (Rena 2017, Nauck 2016).“

New references:

Rena G, Hardie DG, Pearson ER. The mechanisms of action of metformin. Diabetologia. 201760:1577-1585.

Nauck M. Incretin therapies: highlighting common features and differences in the modes of action of glucagon-like peptide-1 receptor agonists and dipeptidyl peptidase-4 inhibitors. Diabetes Obes Metab. 2016;18:203-16.

6) The generalisability of the results to other populations should be discussed.

A: The following text is added to the Discussion section, line 288: “The Finnish population is mostly Caucasian, so generalizability of our results to other ethnicities is uncertain.”

7) The manuscript should highlight the novel aspects of this study compared to previous research.

A: The following text is included to the Discussion: “The novelty of our study is simultaneous evaluation of glycemic status and antidiabetic medication use as prognostic factors of BCa at population level.”

8) The clinical implications of the study findings should be discussed in detail.

A: In our opinion, the most important clinical implication from our study is, that BCa patients could benefit from effective blood glucose management.

We have added the following text 1^st^ paragraph of the Discussion:

“This suggests, that effective follow-up and management of hyperglycemia in BCa patients may provide survival benefits.”

And to the Conclusion:

“Effective management of hyperglycemia is likely beneficial in BCa patients.”

9) The limitations of the study, such as potential sources of bias and the generalisability of the results, should be clearly stated.

A: We have expanded our Discussion (8^th^ and 9^th^ paragraph) on possible confounding by missing information on smoking, limited information on tumor stage and BCa treatments and missing information on lifestyle factors. We now also provide estimates to which direction these limitations may have biased our results.

10) The manuscript should be carefully checked for grammatical errors, spelling mistakes, or unclear presentation.

A: We have performed a language check, and have improved the clarity of presentation to our best ability. We are happy to revise further, if some specific parts of the manuscript remain unclear.

Reviewer #2:

In the submitted article, "Diabetes and Bladder Cancer Prognosis in a Finnish Population-based Cohort," Vuoristo et al. investigate the association between diabetes and bladder cancer prognosis in a Finnish cohort. They find that diabetic hyperglycemia post-diagnosis is linked to increased mortality, although the analysis is limited by insufficient smoking data and incomplete information on glycemic control. However, the study has several other limitations, with comments, suggestions, and concerns that need addressing, and the text in the manuscript requires significant modifications.

Main Concerns:

1. Variable Availability of Comorbidity Data: While diabetes and other comorbidities such as hypertension and obesity were accounted for, the methods section does not specify how consistently these were recorded across the study period. Variations in recording these data can affect the study's ability to accurately assess the impact of these comorbid conditions on bladder cancer outcomes.

A: We have added a clarification of this issue to Methods, Information on diabetes and comorbidities, last paragraph:

“We assessed the risk of comorbidity by using the Charlson comorbidity index (CCI) [15]. Diagnoses for comorbidities were obtained from HILMO and supplemented with medication data from the SII prescription database. These two registers complement each other, as HILMO database records only the diagnoses made in tertiary health care units, thus underestimating the full prevalence of conditions that are mainly managed in primary care, such as hypertension. Prescription database, on the other hand, records all medication purchases regardless of where they have been prescribed, but does not record the indication for prescription.”

2. Suboptimal Control of Confounders: The study utilized multivariable Cox regression models which adjusted for several factors. However, it's unclear if all potential confounders were adequately controlled. For instance, treatment modalities and tumor characteristics could also influence survival outcomes but were not extensively detailed in the context of their influence on the diabetes-cancer link. The author should include detailed analyses of how different treatments and tumor characteristics influence the diabetes-bladder cancer mortality link to provide deeper insights. This would help in understanding whether the observed associations are directly due to diabetes or mediated by other factors.

A: We agree in full. Unfortunately, detailed information on tumor characteristics, such as tumor grade or presence of *carcinoma in situ* lesions, nor detailed information on treatment, such as BCG instillations were not available from population-wide registries used for this study. We used the limited data we had available in the analysis, but is is clear that verification is needed from other study cohorts with more extensive data on tumor characteristics and treatment.

We discuss these limitations in the Discussion:

“FCR registers only tumor extent as local vs. metastasized, whereas no information on muscle invasion or carcinoma in situ was available. Further, information on tumor extent on diagnosis was available only for 74% of the cohort. Limiting the analysis to cases with this information available abolished the risk association between post-diagnostic glucose level and mortality. This suggests that cases with missing information may have caused bias. Furthermore, we did not have information on intravesical treatments, like BCG instillations. However, these are unlikely to differ by anti-DM medication use….. Therefore, our results need to be confirmed in other cohorts with more detailed information on clinical risk factors and comorbidities.”

3. Statistical Significance in Subgroups: Some of the subgroup analyses, such as those involving pre-diagnostic glycemic levels, did not reach statistical significance. This raises questions about the robustness of these associations and whether they could be due to chance. The author should employ advanced statistical techniques that can handle missing data effectively, such as multiple imputation, which might mitigate some of the biases arising from incomplete data. Additionally, using stratified models or propensity score matching could better control for unmeasured confounders.

A: It is true, that our results in general are hypothesis generating and require confirmation from further studies. Multiple imputation methods are uncertain to remove bias, as the imputations are made based on the assumptions of the researchers. Propensity scores are also calculated based on available data, and their value over multivariate adjusted models in large datasets such as ours has been questioned (Hadley J, JNCI 2010). Therefore, the assumptions and calculations for advanced mathematical modeling would require an article of it’s own, which would compare the results obtained with the imputed dataset to those in the present analysis based on recorded data only. In this article we aim to evaluate the impact of missing data in sensitivity analyses limiting the cohort to participants with the data available, as the large size of the cohort allows that in most cases and comparing the results to those using the full dataset.

Hadley J, Yabroff KR, Barrett MJ, Penson DF, Saigal CS, Potosky AL. Comparative effectiveness of prostate cancer treatments: evaluating statistical adjustments for confounding in observational data. J Natl Cancer Inst. 2010 Dec 1;102(23):1780-93. doi: 10.1093/jnci/djq393. Epub 2010 Oct 13. PMID: 20944078; PMCID: PMC2994860.

Text Problems:

1. Original: "Diabetic post-diagnostic blood glucose level was associated with worse BCa-specific HR 1.58 (1.10–2.28) and overall survival HR 1.50 (1.20–1.89)."
Suggestion: "The post-diagnostic blood glucose levels in diabetics were associated with a worse BCa-specific hazard ratio (HR) of 1.58 (1.10–2.28) and an overall survival HR of 1.50 (1.20–1.89)."

A: As defined in the methods, we categorized participants to normoglycemic or hyperglycemic based on their median fasting blood glucose or HbA1c. Hence the expression “Diabetic post-diagnostic blood glucose level”. All of the participants with blood glucose measurements available could have been diabetics, even some of those who had normoglycemia due to good treatment balance. Therefore, we have clarified the sentence as follows:

“Hyperglycemia at diabetic level after BCa diagnosis was associated..”

2. Original: "The risk increase was slightly lower among hyperglycemic DM medication users (HR 1.43 (0,68–3.02) than non-users HR 1.80 (1.13–2.85)."
Suggestion: "The increase in risk was slightly lower among hyperglycemic DM medication users, with an HR of 1.43 (0.68–3.02), compared to non-users, who had an HR of 1.80 (1.13–2.85)."

A: Corrected as suggested.

3. Original: "We assessed the risk of comorbidity by using Charlson comorbidity index (CCI)."
Suggestion: "We assessed the risk of comorbidity using the Charlson Comorbidity Index (CCI)."

A: Corrected as suggested.

4. Original: "Information was collected on fasting glucose and glycated haemoglobins (HbA1c) levels during 1995-2012 for a subset of study population living in the Pirkanmaa region in Finland."
Suggestion: "Information was collected on fasting glucose and glycated hemoglobin (HbA1c) levels from 1995-2012 for a subset of the study population living in the Pirkanmaa region of Finland."

A: Corrected as suggested.

5. Original: "Diagnoses made in 1995 were recorded as ICD-9 codes: 250 participants had diagnosis for diabetes, 272 for hypercholesterolemia, 401 for hypertension and 278 for obesity."
Suggestion: "Diagnoses made in 1995 were recorded using ICD-9 codes: 250 participants were diagnosed with diabetes, 272 with hypercholesterolemia, 401 with hypertension, and 278 with obesity."

A: Corrected as suggested.

Reviewer #3:

The study includes a large cohort of 14,638 BCa patients diagnosed in Finland between 1995 and 2012. Utilizing data from three national databases, the study evaluates the impact of diabetes, measured through blood glucose levels and HbA1c, on BCa-specific and overall mortality using multivariable Cox regression models. The findings indicate that diabetic blood glucose levels are associated with worse BCa-specific and overall survival, with the increased risk being slightly mitigated among those using antidiabetic medication. The study highlights the need for further research to understand the mechanisms behind these associations and the potential implications for clinical management of BCa patients with diabetes.
Here are my questions based on the article:

1. How did the researchers ensure the completeness and accuracy of the data obtained from the three national databases, and what measures were taken to address any potential data gaps or inconsistencies?

A: A text was added to the end of the first paragraph in Materials and methods section, line 73: “The combination of the information from different registries concerning study subjects was possible by using unique personal social security number.”

Inconsistencies due to merging of data from different registries were checked and corrected before the study analysis. Using nationwide registries gave us a very large study cohort, which allowed addressing of data gaps by limiting the cohort to participants with complete information available while still retaining sufficient numbers to perform the analyses.

2. What criteria were used to classify participants' glycemic status, and how did the study account for variations in blood glucose levels and HbA1c measurements over time?

A: We have clarified this in Mat & Met, Information on blood glucose and glycated haemoglobin (HbA1c) levels:

“For each year starting from the year of BCa diagnosis we calculated yearly median for fasting glucose and HbA1c using all the measurements on a given year. For years without available measurements, we used the latest available yearly median. Additionally, we calculated median for the year preceding the year of BCa diagnosis.”

Variation over the follow-up was modelled using time-dependent variables. We describe this in Mat & Met, Statistical analysis:

“Glycemic status after BCa diagnosis was analyzed as time-dependent variable, where the status was updated separately for each follow-up year.”

3. How did the use of antidiabetic medication influence the survival outcomes of BCa patients, and what potential mechanisms might explain the observed differences in mortality risk between medication users and non-users?

A: We did not observe any significant effect modification by antidiabetic medication use, which we report in the Results:

”DM medication did not significantly modify the association between hyperglycemia and BCa mortality: the risk increase by hyperglycemia was slightly less pronounced among DM medication users (HR 1.43 (0,68–3.02)) than non-users (HR 1.80 (1.13–2.85)) nevertheless remaining elevated compared to normoglycemic participants”

As the antidiabetic medication use did not markedly change the results, we did not discuss further any potential mechanisms

4. What confounding factors were considered in the multivariable Cox regression models, and how did the study address potential confounders such as smoking and comorbidities?

A: Multivariable Cox regression analysis was adjusted for age, gender, primary treatment of BCa, tumor extent at diagnosis, hypertension, hypercholesterolemia and CCI. This is described in Mat & Met, Statistical analysis, end of 2^nd^ paragraph.

In order to address confounding due to comorbidities we calculated Charlson comorbidity index (CCI) to be included in the model adjustments.

Unfortunately, smoking is not recorded by Finnish national registries. In a few cases there was a recorded diagnosis of tobacco smoking in HILMO registry, but this was non-systematic and available for a small subpopulation only. We acknowledge this limitation in the Discussion:

“An essential limitation of our dataset is that smoking is not routinely recorded by national registries.”

5. How did the associations between diabetic glycemic status and BCa-specific mortality differ by gender and cancer stage, and what implications do these findings have for personalized treatment strategies in BCa patients with diabetes?

A: Gender did not modify the risk associations between hyperglycemia and bladder cancer specific and overall survival. Therefore, good glycemic control is essential in BCa patients regardless of their gender. This is now mentioned in Disucssion, beginning of the 4^th^ paragraph:

“Based on subgroup analysis, hyperglycemia was a similar risk factor of BCa death both among men and women, suggesting that BCa patients may benefit from hyperglycemia management regardless of their gender.”

Cancer stage, depicted as tumor extent in our data, modified the association wth BCa-specific survival but not with the overall survival. This is described in Results, subgroup analyses, last paragraph:

“Hyperglycemia was associated with worse overall survival similarly regardless of tumor extent at diagnosis. However, tumor extent did modify the association with BCa-specific survival; diabetic blood glucose level was associated with elevated BCa mortality in participants with advanced disease at diagnosis, HR 2.89 (1.33–3.93), but not among those diagnosed with localized disease HR 0.85 (0.42–1.74).”

Thus, our data suggests that especially those with advanced BCa may benefit from effective management of glycemia. We have added these implications into Discussion, paragraphs 4 and 5

Here are the grammatical and phrasing errors found in the provided document along with corrections:
1. Location: Abstract (Lines 21-22)
- Original: "Diabetes might increase a risk of bladder cancer (BCa) incidence and bladder cancer specific death."
- Correction: "Diabetes might increase the risk of bladder cancer (BCa) incidence and bladder cancer-specific death."

A: Corrected as suggested.

2. Location: Abstract (Lines 27-28)
- Original: "Three databases were used to obtain information on BCa, diabetes and comorbidities."
- Correction: "Three databases were used to obtain information on BCa, diabetes, and comorbidities."
A: Corrected as suggested.

3. Location: Abstract (Lines 30-31)
- Original: "During median follow-up of 4.3 years after BCa diagnosis 3,582 (24.5 %) participants died of BCa."
- Correction: "During a median follow-up of 4.3 years after BCa diagnosis, 3,582 (24.5%) participants died of BCa."

A: Corrected as suggested.

4. Location: Abstract (Line 33)
- Original: "The risk increase was slightly lower among hyperglycemic DM medication users (HR 1.43 (0,68–3.02) than non-users HR 1.80 (1.13–2.85)."
- Correction: "The risk increase was slightly lower among hyperglycemic DM medication users (HR 1.43 (0.68–3.02)) than non-users (HR 1.80 (1.13–2.85))."
A: Corrected as suggested.

5. Location: Abstract (Line 37)
- Original: "This was observed in both genders."
- Correction: "This was observed in both men and women."

A: Corrected as suggested.

6. Location: Introduction (Line 43)
- Original: "Bladder cancer (BCa) is the ninth most common cancer worldwide [1]. Well-known risk factors are smoking, male gender, living in developed countries and chemical exposure [1,2]."
- Correction: "Bladder cancer (BCa) is the ninth most common cancer worldwide [1]. Well-known risk factors include smoking, being male, living in developed countries, and chemical exposure [1,2]."

A: Corrected as suggested.

7. Location: Introduction (Line 50)
- Original: "These presumably support growth of cancer cells by offering energy and growth factors for rapid cell growth."
- Correction: "These presumably support the growth of cancer cells by providing energy and growth factors for rapid cell growth."

A: Corrected as suggested.

8. Location: Results (Line 156)
- Original: "No clear differences in Charlson co-morbidity score or prevalence of COPD were observed by medication use or blood glucose level."
- Correction: "No clear differences in Charlson comorbidity score or prevalence of COPD were observed by medication use or blood glucose level."

A: Corrected as suggested.

9. Location: Results (Line 160)
- Original: "DM medication users had similar overall mortality than non-users, while hyperglycemic participants had higher overall mortality than normoglycemic participants."
- Correction: "DM medication users had similar overall mortality to non-users, while hyperglycemic participants had higher overall mortality than normoglycemic participants."

A: Corrected as suggested.

10. Location: Results (Line 174)
- Original: "The risk increase was slightly lower among hyperglycemic DM medication users HR 1.43 (0,68–3.02) than non-users HR 1.80 (1.13–2.85) but remained elevated compared to normoglycemia in both groups."
- Correction: "The risk increase was slightly lower among hyperglycemic DM medication users (HR 1.43 (0.68–3.02)) than non-users (HR 1.80 (1.13–2.85)) but remained elevated compared to normoglycemia in both groups."

A: Corrected as suggested.

11. Location: Discussion (Line 219)
- Original: "Expectedly, also overall mortality was higher in diabetics compared to non-diabetics."
- Correction: "As expected, overall mortality was also higher in diabetics compared to non-diabetics."

A: Corrected as suggested.

12. Location: Discussion (Line 240)
- Original: "Therefore, they might be treated in less aggressive manner."
- Correction: "Therefore, they might be treated in a less aggressive manner."

A: Corrected as suggested.

13. Location: Discussion (Line 258)
- Original: "For our knowledge only one study has previously reported increased risk of BCa-specific death among women with diabetes."
- Correction: "To our knowledge, only one study has previously reported an increased risk of BCa-specific death among women with diabetes."

A: Corrected as suggested.

14. Location: Discussion (Line 265)
- Original: "To decrease this limitation, we adjusted the analysis for records of COPD and smoking from the HILMO database, but the information especially on smoking was severely limited."
- Correction: "To mitigate this limitation, we adjusted the analysis for records of COPD and smoking from the HILMO database, but the information on smoking was particularly limited."

A: Corrected as suggested.

15. Location: Conclusions (Line 281)
- Original: "whereas antidiabetic medication use may mitigate the risk increase to small degree."
- Correction: "whereas antidiabetic medication use may mitigate the risk increase to a small degree."

A: This text was deleted as a suggestion of another reviewer.

Reviewer #4: Although this study is a large-scale investigation, encompassing almost all bladder cancer cases in Finland from 1995 to 2012, and explores the relationship between diabetes and bladder cancer prognosis through the analysis of blood glucose levels and antidiabetic medication history, it has two major limitations that significantly impact its clinical relevance.

Firstly, the data is outdated. The study covers diabetic patients from 1995 to 2012, but many new antidiabetic drugs have been introduced since 2012, such as SGLT-2 inhibitors and GLP-1 receptor agonists. These newer medications can significantly affect patients' weight, blood glucose levels, and even urine glucose, potentially influencing the final risk assessment. Therefore, I recommend the authors use more recent data for their analysis.

A: The focus of this paper is on hyperglycemia as prognostic factor in BCa, antidiabetic medication is studied as a possible effect modificator. Even if our options to manage hyperglycemia have since improved with new drug groups available for treatment, we believe that the main message of our study, that hyperglycemia is dangerous in BCa patients and should be managed effectively, remains relevant to this day.

We are in the process of getting permissions for an updated data. However, the process always takes time, and unfortunately we don’t yet have updated data available.

Secondly, there is a conceptual confusion. Not using antidiabetic drugs does not equate to not having diabetes. In fact, many patients can control their blood glucose levels solely through diet and exercise without needing medication, but this does not change the fact that they have diabetes or that they may have hyperinsulinemia. Thus, relying solely on the use of antidiabetic drugs to classify individuals as diabetic or non-diabetic is clearly inappropriate. Additionally, categorizing groups based on blood glucose levels is also flawed. When patients manage their blood glucose to normal levels through medication, they are still diabetic, their insulin levels have not improved, and insulin resistance remains. They may still be using antidiabetic drugs.

A: We agree in full. We have clarified our terminology throughout the manuscript; instead of using the vague term “diabetics”, we now define our comparison groups as participants with normoglycemia or hyperglycemia, and users or non-users of antidiabetic medication.

Minor issues
The study does not differentiate between type 1 and type 2 diabetes. As the authors mentioned from Line49-51, elevated blood glucose and high insulin levels are risk factors, and the fundamental difference between type 1 and type 2 diabetes is insulin levels. If the two types cannot be distinguished, using blood glucose alone to assess the risk of diabetes for cancer is inappropriate. Although this was explained in the limitations section, I suggest focusing the analysis on only one type of diabetes.

A: Unfortunately, we did not have information on participants’ insulin levels, thus cannot make a distinction between type 1 and 2 diabetes with any certainty.

Additionally, the authors did not assess the impact of smoking, which is a significant risk factor for bladder cancer. The proportion of smokers is high among diabetic patients, which affects the accuracy of the conclusions. This should be further explained in the limitations section.

A: True. Unfortunately, information on smoking is not recorded by the national registries used in our study. We acknowledge this limitation, as well as it’s potential impact on our results in the Discussion:

“An essential limitation of our dataset is that smoking is not routinely recorded by national registries. Smoking may have confounded the results, likely raising the observed BCa mortality among anti-DM medication users, if smoking was more common among them.”

The study concludes that antidiabetic medications may have a protective effect in reducing the risk of bladder cancer. However, since the specific medication regimens of the patients are unclear, this conclusion is not accurate. In particular, the potential risk of bladder cancer associated with pioglitazone has garnered attention from various organizations, including the FDA, in recent years.

A: We agree. The conclusion has been revised:

“Hyperglycemia is associated with elevated BCa-specific mortality. Effective management of hyperglycemia is likely beneficial in BCa patients.”

Why was acarbose excluded?

A: Acarbose is very rarely used in Finland, there were not enough users to be included in the analysis.

There are some grammatical and spelling errors in the articles, such as
Line 53-55, I suggest using “According to a meta-analysis of 36 studies, diabetes was associated with an increased risk of bladder cancer (BCa) in men【6】. A prospective population-based cohort study also found an increased risk of BCa death, but only in men【7】. Conversely, another study with 1,000 patients found no association between diabetes (type 1 or 2) and BCa risk or mortality【8】.”

A: Corrected as suggested.

Line67-69, I suggest using “The most common tumor morphologies were transitional cell carcinoma(12,027 cases, 82.0%), papillary transitional cell carcinoma( 1,626 cases ,11.1%), and unspecified malignant neoplasm( 533 cases ,3.6%).

A: Corrected as suggested.
