# Supplementary material for: Hyperglycemia and bladder cancer prognosis in a Finnish population-based cohort
Source: PLoS One. 2025 Dec 12;20(12):e0338215. doi: 10.1371/journal.pone.0338215 (PMC12700417; doi:10.1371/journal.pone.0338215)
Supplement: Attachment — Submitted filename: Response_to_Reviewers_auresp_3.docx [file pone.0338215.s005.docx]

PONE-D-24-16036R2
Hyperglycemia and bladder cancer prognosis in a Finnish population-based cohort
PLOS ONE

Dear editor,
Thank You for possibility to answer these questions. We have thoroughly considered our answers to reviewer´s remarks and submit answers here and the revised version has been made with and without mark changed as instructed. We hope that after these corrections, our manuscript could be worth of publishing in PLOS ONE.

Journal Requirements:

Please review your reference list to ensure that it is complete and correct. If you have cited papers that have been retracted, please include the rationale for doing so in the manuscript text, or remove these references and replace them with relevant current references. Any changes to the reference list should be mentioned in the rebuttal letter that accompanies your revised manuscript. If you need to cite a retracted article, indicate the article’s retracted status in the References list and also include a citation and full reference for the retraction notice.

RESPONSE: The reference list has been reviewed. One reference was changed to more proper one and three references were deleted. The style of reference list was changed to match PLOS ONE style.

1. Antoni S, Ferlay J, Soerjomataram I, Znaor A, Jemal A, Bray F. Bladder Cancer Incidence and Mortality: A Global Overview and Recent Trends. Eur Urol. 2017;71~~(1)~~: 96-108.
2. Cumberbatch MGK, Jubber I, Black PC, Esperto F, Figueroa JD, Kamat AM, et al. Epidemiology of Bladder Cancer: A Systematic Review and Contemporary Update of Risk Factors in 2018. Eur Urol. 2018;74~~(6)~~: 784-795.
3. Dobruch J, Daneshmand S, Fisch M, Lotan Y, Noon AP, Resnick MJ, et al. Gender and Bladder Cancer: A Collaborative Review of Etiology, Biology, and Outcomes. Eur Urol. 2016;69~~(2)~~: 300-310.
4. Larsson SC, Orsini N, Brismar K, Wolk A~~et al~~. Diabetes mellitus and risk of bladder cancer: a meta-analysis. Diabetologia. 2006;49~~(12)~~: 2819-2823.
5. Zhou XH, Qiao Q, Zethelius B, Pyörälä K, Söderberg S, Pajak A, et al. Diabetes, prediabetes and cancer mortality. Diabetologia. 2010;53~~(9)~~: 1867-1876.
6. Zhu Z, Wang X, Shen Z, Lu Y, Zhong S, Xu C. et al. Risk of bladder cancer in patients with diabetes mellitus: an updated meta-analysis of 36 observational studies. BMC Cancer. 2013;13: 310. doi: 10.1186/1471-2407-13-310.
7. Coughlin SS, Calle EE, Teras LR, Petrelli J, Thun MJ. ~~et al.~~ Diabetes Mellitus as a Predictor of Cancer Mortality in a Large Cohort of US Adults. Am J Epidemiol. 2004;159~~(12)~~: 1160-1167.
8. Goossens ME, Zeegers MP, Bazelier MT, De Bruin ML, Buntinx F, de Vries F. ~~et al.~~ Risk of bladder cancer in patients with diabetes: a retrospective cohort study. BMJ Open. 2015;5~~(6)~~: e007470. doi: 10.1136/bmjopen-2014-007470.
9. Teppo L, Pukkala E, Lehtonen M. Data Quality and Quality Control of a Population-Based Cancer Registry: Experience in Finland. Acta Oncol. 1994;33~~(4)~~: 365-369.
10. ~~Finnish Cancer Registry. http://cancerregistry.fi/~~
11. Sund R. Quality of the Finnish Hospital Discharge Register: A systematic review. Scand J Public Health. 2012;40~~(6)~~: 505-515.
12. Klaukka T. The Finnish database on drug utilisation. Norwegian Journal of Epidemiology. 2001;11~~(1)~~:19-22.
13. ~~Finnish National Health Care Insurance. https://www.kela.fi/web/en/national-health-insurance~~
14. ~~The Anatomical Therapeutic Chemical (ATC) classification system. https://www.whocc.no/atc_ddd_index/~~
15. Charlson ME, Carrozzino D, Guidi J, Patierno C. Charlson Comorbidity Index: A Critical Review of Clinimetric Properties. Psychother Psychosom. 2022;91: 8-35. ~~Peterson J, Paget S, Lachs M, et al. The risk of comorbidity.~~ *~~Ann Rheum Dis~~*~~. 2012;71(5):635-637.~~
16. Campbell PT, Newton CC, Patel AV, Jacobs EJ, Gapstur SM. ~~et al.~~ Diabetes and Cause-Specific Mortality in a Prospective Cohort of One Million U.S. Adults. Diabetes Care. 2012;35~~(9)~~: 1835-1844.
17. Dong L~~v~~, Ying X~~iang~~, Tao S~~ong~~, Guang-Peng Z, Tai-Ming S. ~~et al.~~ Diabetes Is a Risk Factor for the Prognosis of Patients with Bladder Cancer: A Meta-Analysis. J Oncol. 2022; 1997507. doi: 10.1155/2022/1997507.
18. Van de Poll-Franse LV, Houterman S, Janssen-Heijnen MLG, Dercksen MW Coebergh JWW, Haak HR. ~~et al.~~ Less aggressive treatment and worse overall survival in cancer patients with diabetes: A large population based analysis. Int J Cancer. 2007;120~~(9)~~: 1986-1992.
19. Faiena I, Dombrovskiy VY, Sultan RC, Salmasi AH, Singer EA, Weiss RE. ~~et al.~~ Effect of Uncontrolled Diabetes on Outcomes After Cystectomy in Patients With Bladder Cancer: A Population-Based Study. Clin Genitourin Cancer. 2016;14~~(5)~~: e509-e514.
20. Oh JJ, Kang MY, Jo JK, Lee HM, Byun SS, Lee SE et al. Association between diabetes mellitus and oncological outcomes in bladder cancer patients undergoing radical cystectomy. Int J Urol. 2015;22~~(12)~~: 1112-1117.
21. Tseng CH. Diabetes and risk of bladder cancer: a study using the National Health Insurance database in Taiwan. Diabetologia. 2011;54~~(8)~~: 2009-2015.
22. Tripathi A, Folsom AR, Anderson KE. Risk factors for urinary bladder carcinoma in postmenopausal women. Cancer. 2002;95~~(11)~~: 2316–2323.

[Note: HTML markup is below. Please do not edit.]

Reviewers' comments:

Reviewer's Responses to Questions

Comments to the Author

1. If the authors have adequately addressed your comments raised in a previous round of review and you feel that this manuscript is now acceptable for publication, you may indicate that here to bypass the “Comments to the Author” section, enter your conflict of interest statement in the “Confidential to Editor” section, and submit your "Accept" recommendation.

Reviewer #3: All comments have been addressed

Reviewer #5: All comments have been addressed

RESPONSE: No actions needed

________________________________

2. Is the manuscript technically sound, and do the data support the conclusions?

The manuscript must describe a technically sound piece of scientific research with data that supports the conclusions. Experiments must have been conducted rigorously, with appropriate controls, replication, and sample sizes. The conclusions must be drawn appropriately based on the data presented.

Reviewer #3: Yes

Reviewer #5: (No Response)

RESPONSE: No actions needed

________________________________

3. Has the statistical analysis been performed appropriately and rigorously?

Reviewer #3: Yes

Reviewer #5: (No Response)

RESPONSE: No actions needed

________________________________

4. Have the authors made all data underlying the findings in their manuscript fully available?

The PLOS Data policy<<http://track.editorialmanager.com/CL0/http:%2F%2Fwww.plosone.org%2Fstatic%2Fpolicies.action%23sharing/1/010f0195c693853b-e9391df4-831f-4c09-bc3d-cab85d5402cd-000000/59fVLGzW1aiED_EG1p4o-5qRD0lEkysVgQBquo65xh8=203>> requires authors to make all data underlying the findings described in their manuscript fully available without restriction, with rare exception (please refer to the Data Availability Statement in the manuscript PDF file). The data should be provided as part of the manuscript or its supporting information, or deposited to a public repository. For example, in addition to summary statistics, the data points behind means, medians and variance measures should be available. If there are restrictions on publicly sharing data—e.g. participant privacy or use of data from a third party—those must be specified.

Reviewer #3: Yes

Reviewer #5: (No Response)

RESPONSE: No actions needed

________________________________

5. Is the manuscript presented in an intelligible fashion and written in standard English?

PLOS ONE does not copyedit accepted manuscripts, so the language in submitted articles must be clear, correct, and unambiguous. Any typographical or grammatical errors should be corrected at revision, so please note any specific errors here.

Reviewer #3: Yes

Reviewer #5: (No Response)

RESPONSE: No actions needed

________________________________

6. Review Comments to the Author

Please use the space provided to explain your answers to the questions above. You may also include additional comments for the author, including concerns about dual publication, research ethics, or publication ethics. (Please upload your review as an attachment if it exceeds 20,000 characters)

Reviewer #3: The authors have thoroughly addressed all of my concerns. I have no further questions and recommend proceeding with the acceptance process according to the journal's guidelines.

RESPONSE: Thank You.

Reviewer #5: In this study, the authors investigate the potential association between diabetes and bladder cancer prognosis.
I think the revisions made by the authors in line with the referee suggestions are sufficient. I have no additional questions about the study. However, while correcting the grammar of the article, I think some words were forgotten to be deleted in the 3rd paragraph of the introduction.

RESPONSE: Thank You. The 3^rd^ paragraph of the introduction has been checked and corrected.

________________________________

7. PLOS authors have the option to publish the peer review history of their article (what does this mean?<<https://track.editorialmanager.com/CL0/https:%2F%2Fjournals.plos.org%2Fplosone%2Fs%2Feditorial-and-peer-review-process%23loc-peer-review-history/1/010f0195c693853b-e9391df4-831f-4c09-bc3d-cab85d5402cd-000000/rZ0UH0RJsU11eawvF74c2CZpE-uit5dx4NNTYvRn_Ds=203>>). If published, this will include your full peer review and any attached files.


If you choose “no”, your identity will remain anonymous but your review may still be made public.


Do you want your identity to be public for this peer review? For information about this choice, including consent withdrawal, please see our Privacy Policy<<https://track.editorialmanager.com/CL0/https:%2F%2Fwww.plos.org%2Fprivacy-policy/1/010f0195c693853b-e9391df4-831f-4c09-bc3d-cab85d5402cd-000000/mQPv1YvGfC3vtt3IRaxLvp4w_K9jYXiU5k7nIJEg8mM=203>>.

Reviewer #3: Yes: peng wang

Reviewer #5: No
