# Supplementary material for: Hyperglycemia and bladder cancer prognosis in a Finnish population-based cohort
Source: PLoS One. 2025 Dec 12;20(12):e0338215. doi: 10.1371/journal.pone.0338215 (PMC12700417; doi:10.1371/journal.pone.0338215)
Supplement: Attachment — Submitted filename: Response_to_Reviewers_auresp_5.docx [file pone.0338215.s007.docx]

PONE-D-24-16036R2
Hyperglycemia and bladder cancer prognosis in a Finnish population-based cohort
PLOS ONE

Dear editor,
Thank You for possibility to answer these questions. We have thoroughly considered our answers to reviewer´s remarks and submit answers here and the revised version has been made with and without mark changed as instructed. We hope that after these corrections, our manuscript could be worth of publishing in PLOS ONE.

Journal Requirements:

Please review your reference list to ensure that it is complete and correct. If you have cited papers that have been retracted, please include the rationale for doing so in the manuscript text, or remove these references and replace them with relevant current references. Any changes to the reference list should be mentioned in the rebuttal letter that accompanies your revised manuscript. If you need to cite a retracted article, indicate the article’s retracted status in the References list and also include a citation and full reference for the retraction notice.

RESPONSE: Reference list has been checked and one reference was added based on reviewer´s comment.

12. WHO Collaborating Centre for Drug Statistics Methodology,

Guidelines for ATC classification and DDD assignment 2024. Oslo, Norway, 2023. ISBN 978-82-8406-428-4


[Note: HTML markup is below. Please do not edit.]

Reviewers' comments:

Reviewer's Responses to Questions

Comments to the Author

1. If the authors have adequately addressed your comments raised in a previous round of review and you feel that this manuscript is now acceptable for publication, you may indicate that here to bypass the “Comments to the Author” section, enter your conflict of interest statement in the “Confidential to Editor” section, and submit your "Accept" recommendation.

Reviewer #3: All comments have been addressed

Reviewer #5: (No Response)


6. Review Comments to the Author

Please use the space provided to explain your answers to the questions above. You may also include additional comments for the author, including concerns about dual publication, research ethics, or publication ethics. (Please upload your review as an attachment if it exceeds 20,000 characters)

Reviewer #3: The authors have thoroughly addressed all of my concerns. I have no further questions and recommend proceeding with the acceptance process according to the journal's guidelines.
RESPONSE: Thank you.

Reviewer #5: I want to thank the authors for the revisions made to the article. (Location: Materials and methods, Lines 98-99 ) The authors removed the 14th reference (‘’14. The Anatomical Therapeutic Chemical (ATC) classification system. <https://www.whocc.no/atc_ddd_index/> ‘’) from the article, but the referenced text was not removed, and no reference was provided. I recommend that this situation be explained, or that the appropriate reference be added.
RESPONSE: This is the relevant comment and we have added the appropriate published reference for this text, instead of a website:

12	WHO Collaborating Centre for Drug Statistics Methodology,

Guidelines for ATC classification and DDD assignment 2024. Oslo, Norway, 2023. ISBN 978-82-8406-428-4

________________________________
