# Supplementary material for: Hyperglycemia and bladder cancer prognosis in a Finnish population-based cohort
Source: PLoS One. 2025 Dec 12;20(12):e0338215. doi: 10.1371/journal.pone.0338215 (PMC12700417; doi:10.1371/journal.pone.0338215)
Supplement: Attachment — Submitted filename: Response_to_Reviewers_auresp_6.docx [file pone.0338215.s008.docx]

PONE-D-24-16036R2
Hyperglycemia and bladder cancer prognosis in a Finnish population-based cohort
PLOS ONE

Dear editor,
Thank You for possibility to answer these questions. We have thoroughly considered our answers to reviewer´s remarks and submit answers here and the revised version has been made with and without mark changed as instructed. We hope that after these corrections, our manuscript could be worth of publishing in PLOS ONE.

6. Review Comments to the Author

Please use the space provided to explain your answers to the questions above. You may also include additional comments for the author, including concerns about dual publication, research ethics, or publication ethics. (Please upload your review as an attachment if it exceeds 20,000 characters)

Reviewer #3: The authors have thoroughly addressed all of my concerns. I have no further questions and recommend proceeding with the acceptance process according to the journal's guidelines.

RESPONSE: Thank you for the helpful review.

Reviewer #5: I want to thank the authors for their thorough consideration and revision of all my concerns. The reference numbers have been updated following the revisions to the article. I believe some of the previously changed reference numbers were not removed from the text, so I recommend checking. (Example: Materials and methods, Lines 100, ‘’We assessed the risk of comorbidity by using Charlson comorbidity index (CCI) [1213].’’) I have no additional questions or suggestions, and I recommend proceeding with the acceptance process according to the journal's guidelines.

RESPONSE: Thank you for the helpful review. We have now checked the reference numbers and removed any redundant ones from the text.
