# Supplementary material for: Hyperglycemia and bladder cancer prognosis in a Finnish population-based cohort
Source: PLoS One. 2025 Dec 12;20(12):e0338215. doi: 10.1371/journal.pone.0338215 (PMC12700417; doi:10.1371/journal.pone.0338215)
Supplement: Attachment — Submitted filename: Response_to_Reviewers_auresp_7.docx [file pone.0338215.s009.docx]

PONE-D-24-16036R2
Hyperglycemia and bladder cancer prognosis in a Finnish population-based cohort
PLOS ONE

Dear editor,
Thank You for possibility to answer these questions. We have thoroughly considered our answers to reviewer´s remarks and submit answers here and the revised version has been made with and without mark changed as instructed. We hope that after these corrections, our manuscript could be worth of publishing in PLOS ONE.

Journal Requirements:

If the reviewer comments include a recommendation to cite specific previously published works, please review and evaluate these publications to determine whether they are relevant and should be cited. There is no requirement to cite these works unless the editor has indicated otherwise.

 RESPONSE: We did not receive such recommendations

Please review your reference list to ensure that it is complete and correct. If you have cited papers that have been retracted, please include the rationale for doing so in the manuscript text, or remove these references and replace them with relevant current references. Any changes to the reference list should be mentioned in the rebuttal letter that accompanies your revised manuscript. If you need to cite a retracted article, indicate the article’s retracted status in the References list and also include a citation and full reference for the retraction notice.

RESPONSE: Reference list has been checked

[Note: HTML markup is below. Please do not edit.]

Reviewers' comments:

Reviewer's Responses to Questions

**Comments to the Author**

1. If the authors have adequately addressed your comments raised in a previous round of review and you feel that this manuscript is now acceptable for publication, you may indicate that here to bypass the “Comments to the Author” section, enter your conflict of interest statement in the “Confidential to Editor” section, and submit your "Accept" recommendation.

Reviewer #5: All comments have been addressed

Reviewer #6: (No Response)

2. Is the manuscript technically sound, and do the data support the conclusions?

The manuscript must describe a technically sound piece of scientific research with data that supports the conclusions. Experiments must have been conducted rigorously, with appropriate controls, replication, and sample sizes. The conclusions must be drawn appropriately based on the data presented.

Reviewer #5: Yes

Reviewer #6: Yes

3. Has the statistical analysis been performed appropriately and rigorously?

Reviewer #5: I Don't Know

Reviewer #6: Yes

4. Have the authors made all data underlying the findings in their manuscript fully available?

The [PLOS Data policy](http://track.editorialmanager.com/CL0/http:%2F%2Fwww.plosone.org%2Fstatic%2Fpolicies.action%23sharing/1/010f0198738f10eb-782acb2c-30ca-4cb9-a152-2034c29de5b6-000000/MHdexP6IDkVFDFyMdUObfYu8G_a413b9gkGFr9d29zU=222) requires authors to make all data underlying the findings described in their manuscript fully available without restriction, with rare exception (please refer to the Data Availability Statement in the manuscript PDF file). The data should be provided as part of the manuscript or its supporting information, or deposited to a public repository. For example, in addition to summary statistics, the data points behind means, medians and variance measures should be available. If there are restrictions on publicly sharing data—e.g. participant privacy or use of data from a third party—those must be specified.

Reviewer #5: No

Reviewer #6: No

5. Is the manuscript presented in an intelligible fashion and written in standard English?

PLOS ONE does not copyedit accepted manuscripts, so the language in submitted articles must be clear, correct, and unambiguous. Any typographical or grammatical errors should be corrected at revision, so please note any specific errors here.

Reviewer #5: Yes

Reviewer #6: Yes

6. Review Comments to the Author

Please use the space provided to explain your answers to the questions above. You may also include additional comments for the author, including concerns about dual publication, research ethics, or publication ethics. (Please upload your review as an attachment if it exceeds 20,000 characters)

Reviewer #5: I want to thank the authors for their thorough consideration and revision of all my concerns. I have no additional questions or suggestions, and I recommend proceeding with the acceptance process according to the journal's guidelines.

Reviewer #6: The manuscript is technically sound. The research question is clear and well-defined, the study design (a large, population-based cohort) is appropriate, and the conclusions are well-supported by the data. The statistical methodology, including the use of multivariable Cox regression and time-varying covariates, is appropriate for the research question and data structure.

Strengths:
Large, nationally representative cohort (n=14,638).
Linkage of multiple reliable registries enhances data validity.
Sensitivity and subgroup analyses (including competing risks) strengthen the robustness of findings.
Thoughtful discussion of limitations and contextualization within existing literature.

The statistical analysis is largely rigorous and appropriate. The use of multivariable Cox regression and time-dependent covariates is commendable. The authors correctly adjusted for relevant covariates and provided person-time denominators when reporting mortality rates.

Missing data on tumor stage and primary treatment (available for only 74% and 80%, respectively) were not imputed. Although a complete-case sensitivity analysis was conducted, use of multiple imputation would strengthen the conclusions.

RESPONSE: We see the point. In our opinion, however, data imputation for such large portion of the data, 20-26%, also risks introducing bias based on the assumptions of the imputation. This is the reason we rather did a complete-case analysis. For do mention the limited information on stage as study limitation in the Discussion. We also give recommendation for further studies:

The FCR registers only tumor extent as local vs. metastasized, whereas no information on muscle invasion or carcinoma in situ was available. Further, information on tumor extent on diagnosis was available only for 74% of the cohort. Limiting the analysis to cases with this information available abolished the risk association between post-diagnostic glucose level and mortality. This suggests that cases with missing information may cause bias in our results. Our results require confirmation from datasets with more comprehensive information on stage, possibly via use of multiple imputations.

Dichotomization of continuous glycemic variables (e.g., glucose, HbA1c) may reduce statistical power. Consider exploring dose-response relationships or using categories aligned with clinical thresholds in future work.

RESPONSE: The categories for glycemic variables were indeed stratified by clinical thresholds: 7 mmol/l for fasting plasma glucose (international definition for diabetes mellitus is fasting plasma glucose ≥ 7,0 mmol/l. For HbA1c 53 mmol/mol was the threshold for diabetes at the time of the study period, although it has been lowered since.

We now clarify this in the text, lines 107-110:

“Men were defined as normoglycaemic, or diabetic separately for each follow-up years based on local and international thresholds (<53 to ≥53 mmol/mol, and ≤7 to >7 mmol/l) for yearly mean HbA1c or median fasting glucose, respectively”

I recommend the manuscript be accepted after minor revisions or clarifications, particularly regarding the impact of missing data and the potential use of multiple imputation for future studies.

RESPONSE: Thank you for your constructive comments.

7. PLOS authors have the option to publish the peer review history of their article ([what does this mean?](https://track.editorialmanager.com/CL0/https:%2F%2Fjournals.plos.org%2Fplosone%2Fs%2Feditorial-and-peer-review-process%23loc-peer-review-history/1/010f0198738f10eb-782acb2c-30ca-4cb9-a152-2034c29de5b6-000000/QcnpSssIIUcfLm8iWhrrurznEGye6ti37SFXiFZfFT8=222)). If published, this will include your full peer review and any attached files.


If you choose “no”, your identity will remain anonymous but your review may still be made public.


**Do you want your identity to be public for this peer review?** For information about this choice, including consent withdrawal, please see our [Privacy Policy](https://track.editorialmanager.com/CL0/https:%2F%2Fwww.plos.org%2Fprivacy-policy/1/010f0198738f10eb-782acb2c-30ca-4cb9-a152-2034c29de5b6-000000/AVDkMnHvgJv87Fhly6ohRaxqtFln0Df7TYNMdyXbJrw=222).

Reviewer #5: No

Reviewer #6: No
